# Investigating the role of beta and gamma tACS in visual processing and conscious perception

**DOI:** 10.1093/nc/niaf056

**Published:** 2026-01-19

**Authors:** Yayla A Ilksoy, Alethia de la Fuente, Jacobo Diego Sitt, Enzo Tagliazucchi, Carla Pallavicini

**Affiliations:** Department of Physics, University of Buenos Aires, Pabellón I, Ciudad Universitaria (1428) - C.A.B.A -Argentina; Department of Physics, University of Buenos Aires, Pabellón I, Ciudad Universitaria (1428) - C.A.B.A -Argentina; National Scientific and Technical Research Council, Godoy Cruz 2290 (1425), C.A.B.A., Argentina; Institute of Cognitive and Translational Neuroscience, INECO Foundation, Favaloro University, Marcelo Torcuato de Alvear 1632 (C1021), C.A.B.A., Argentina; Uruguayan Center for Molecular Imaging, CUDIM. Av. Dr. Américo Ricaldoni 2010 (11600) Montevideo, Departamento de Montevideo, Uruguay; Institut du Cerveau - Paris Brain Institute - ICM, INSERM, CNRS, Sorbonne Université, 47 boulevard de l'Hôpital, (F-75013) Paris, France; Department of Physics, University of Buenos Aires, Pabellón I, Ciudad Universitaria (1428) - C.A.B.A -Argentina; National Scientific and Technical Research Council, Godoy Cruz 2290 (1425), C.A.B.A., Argentina; Latin American Brain Health Institute (BrainLat), Universidad Adolfo Ibanez, Av. Diag. Las Torres 2640, 7941169 Santiago, Peñalolén, Región Metropolitana, Chile; Department of Physics, University of Buenos Aires, Pabellón I, Ciudad Universitaria (1428) - C.A.B.A -Argentina; National Scientific and Technical Research Council, Godoy Cruz 2290 (1425), C.A.B.A., Argentina; Cognitive Neuroanatomy Lab, INCC UMR 8002, CNRS, Université Paris Cité, 45 Rue des Saints-Pères, 75006 Paris, France

**Keywords:** tACS, EEG, beta oscillations, gamma oscillations, conscious perception, neural entrainment, visual processing

## Abstract

It has been proposed that both conscious and unconscious perception are associated with a feedforward sweep of oscillatory activity in the gamma band (>40 Hz), while conscious perception also requires recurrent feedback *via* beta band ($\sim$20 Hz) oscillations. To investigate the causal relationship between these oscillations and (un)conscious visual perception, we assessed the effect of transcranial alternating current stimulation (tACS) in the gamma (40 Hz) and beta (20 Hz) bands on the objective and subjective visibility of targets in a metacontrast backward masking task. To capture different aspects of visual experience, we measured *objective visibility*: participants’ ability to correctly categorize the masked stimulus, and *subjective visibility*: participants’ self-report of whether they consciously perceived the stimulus. We expected that 40hz-tACS would affect both the objective visibility and subjective visibility. Moreover, we expected that 20 Hz-tACS would selectively affect the subjective visibility. Our results showed that target visibility was selectively compromised by 20 Hz-tACS but, in contrast to our hypothesis, this effect was specific to objective visibility. Although the power of local beta oscillations increased after 20 Hz-tACS, inter-areal beta synchrony could have nevertheless been impaired, a possibility that should be investigated in the future by means of source reconstructed high density electroencephalography recordings. In summary, our findings suggest that 20 Hz tACS may modulate target visibility, indicating a potential relationship between beta-band activity and visual perception. Future studies could build upon this result by investigating other forms of stimulation and other model organisms, further contributing to our knowledge of how conscious access causally depends on brain oscillations.

## Introduction

Less than a century ago, consciousness was largely considered a philosophical concept beyond empirical research. However, neuroscientific studies on consciousness surged in recent decades, driven by new technologies and experimental paradigms (Crick and Koch 1990; Seth et al. 2008; Tagliazucchi 2020). Research often distinguishes between conscious states (e.g. sleep, coma, and anesthesia) and conscious information access, related to perceptual awareness. Following Crick and Koch’s (Crick and Koch 1990) proposal, most studies on neural correlates of conscious perception focus on vision due to its richness and the advanced understanding of the primate visual system. Visual awareness is commonly studied using paradigms that dissociate objective measures (i.e. the accuracy of identifying or categorizing a stimulus) from subjective reports (i.e. the participant’s conscious experience of seeing the stimulus) (Tagliazucchi 2020). This distinction is crucial, as a stimulus can be processed to support accurate behavioral responses without reaching full conscious awareness (Crick and Koch 1990; Tagliazucchi 2020). In the context of masking, e.g. participants may sometimes categorize a stimulus correctly without reporting that they consciously perceived it, a phenomenon often associated with “blindsight-like” behavior.

To interpret such dissociations, theoretical models of conscious perception are essential. The global workspace (GW) theory equates consciousness with the global availability of information for its subsequence processing by multiple independent and parallel systems that implement specific cognitive functions (Dehaene et al. 2006). This theory provides a theoretical framework for the interpretation of neurophysiological observations related to conscious information access. According to this theory, the reportability of sensory percepts is the result of widespread communication (“broadcasting”). Strong, attended stimuli ignite frontal, parietal, and temporal regions (Dehaene and Naccache 2001; Baars 2002; 2005; Dehaene et al. 2006; 2011; Baars et al. 2013; Tagliazucchi 2020). A reportable stimulus first activates primary sensory areas before propagating to higher-order regions involved in cognition, while nonreportable stimuli remain confined to early sensory areas (Dehaene et al. 2006). Event-related potential analyses reveal that unconscious visual processing occurs early (<270 ms), while conscious perception corresponds to later potentials (>270 ms) involving a broad fronto-parieto-temporal network (Del Cul et al. 2007a; Melloni et al. 2007a). Strong but unattended stimuli may become preconscious, capable of reaching the GW but requiring top-down attention to do so within a short time window (Dehaene et al. 1998a; 2001a; Naccache and Dehaene 2001a; van Gaal et al. 2014a; Tagliazucchi 2020). Lamme (Lamme and Roelfsema 2000; Lamme 2018) proposed that recurrent feedback is crucial for conscious awareness, distinguishing it from unconscious processing, which relies on feedforward activity. They suggested that unattended feedforward activity can extend beyond sensory areas and influence behavior without awareness, aligning with the GW theory’s preconscious states (Dehaene et al. 1998b; 2001b; Naccache and Dehaene 2001b; van Gaal et al. 2014b). This is exemplified by blindsight, where patients correctly categorize visual stimuli without conscious perception. According to their hypothesis, recurrent feedback enhances top-down amplification of sensory signals (Sergent and Dehaene 2004a).

However, GW is just one among several theories that aim to explain the neural mechanisms underlying conscious perception, and many others also emphasize the critical role of feedback processing. For instance, Recurrent Processing Theory (RPT) proposes that consciousness emerges when neural activity becomes locally recurrent within sensory cortices, as opposed to purely feedforward processing (Lamme and Roelfsema 2000; Lamme 2018). In this view, recurrent loops within visual areas are sufficient for the generation of phenomenal consciousness, even without global access or attentional amplification. Lamme further delineates four stages of visual processing based on the presence or absence of attention and recurrent activity, suggesting that conscious experience can occur in the absence of reportability when stimuli are processed in a recurrent but unattended manner (Lamme 2018). This framework contrasts with GW, which equates conscious access with widespread broadcasting and emphasizes reportability as a criterion for consciousness. Yet despite these differences, both theories converge on the idea that recurrent feedback, whether local or global, is essential for conscious perception (Tagliazucchi 2020). Indeed, most contemporary models agree that purely feedforward activity is insufficient to support awareness, and that some form of top-down or recurrent signal is required to amplify and stabilize perceptual content.

Building on this theoretical foundation, recent research has explored how specific neural oscillations, particularly in the gamma and beta frequency bands, may implement the feedback processes implicated in conscious perception. Gamma oscillations have often been associated with conscious information access (Sergent and Dehaene 2004b; Melloni et al. 2007b; Steinmann et al. 2014; Cavinato et al. 2015). However, Gaillard et al. (Gaillard et al. 2009a) observed increased beta synchrony rather than gamma during unmasked *vs*, masked stimuli presentation in epileptic patients with intracranial EEG. Moreover, this activity pattern was restricted to late processing (300–500 ms) and was recurrent in the case of conscious information access, in line with the proposal by Roelfsema & Lamme. Similarly (Lamme and Roelfsema 2000; Lamme 2018), Bastos *et al.* (Bastos et al. 2015a) showed that feedforward activity in the visual cortex is carried by gamma oscillations, while feedback influences depend on beta oscillations. Interestingly, microstimulation of lower visual areas in monkeys induces gamma-oscillations in higher visual areas, while communication in the opposite direction was governed by alpha frequencies (van Kerkoerle et al. 2014a). Although our study focuses on beta and gamma, we reference alpha oscillations to situate our approach within this broader frequency-based framework for understanding directional communication in the brain.

Overall, these findings suggest that subliminal or preconscious information processing occurs *via* a fast feedforward sweep, involving local activity in the gamma range that quickly fades away. In contrast, conscious processing requires feedback influences of distant brain areas carried by beta oscillations. However, most supporting evidence are either or based on animal models, leaving open the question of whether disrupting beta feedback selectively impairs conscious access. Non-invasive electrical stimulation is a suitable method to investigate this causal link in humans. In particular, transcranial alternating current stimulation (tACS) applies a sinusoidal current in a chosen frequency and is capable of modulating neural oscillations at the stimulation frequency. We applied tACS with stimulation frequencies in the gamma (40 Hz) and beta (20 Hz) bands to participants performing a metacontrast backward-masking task. Here, we use the term objective visibility to refer to correct categorization performance, and subjective visibility to refer to the participant’s conscious report of having seen the stimulus. We consistently apply this terminology throughout the manuscript to clarify the dissociation between unconscious and conscious processing pathways and to examine how tACS might differentially influence each. We expected that gamma-tACS would affect objective visibility and subjective visibility; in contrast, we hypothesized that beta-tACS would impact only on subjective visibility, as it would interfere with the recurrent synchronization implicated with conscious access.

## Materials and methods

### Participants

Thirty-one right-handed healthy participants (eight women, 23 men; mean age: 30.39 ± 5.13, range: 22–41) were included. Both participants and researchers were blind to the stimulation condition. All had normal or corrected vision, no neurological history, and were not on medication affecting cortical excitation or had implants (pacemaker, defibrillator, or intracranial electrodes). Written informed consent was obtained per the Helsinki Declaration. The study was approved by the ethics committee of Hospital General de Agudos José María Ramos Mejía (Buenos Aires, Argentina) and conducted following international medical research principles. Participants received no compensation.

### Experimental task and performance

**Figure 1:**
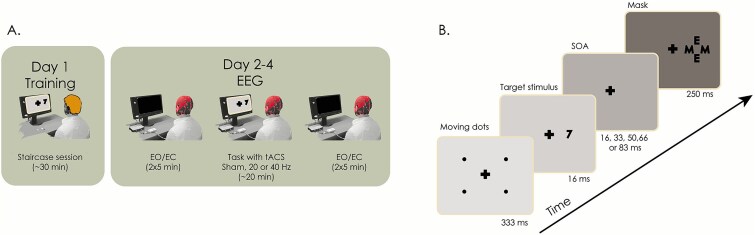
Task set-up and experimental procedure. (A) In total, participants performed four sessions on four nonconsecutive days. The first session included a staircase procedure to determine the appropriate target contrast for that participant. The remaining three sessions were identical in terms of the performed task, but were conducted under different tACS stimulation conditions (sham, 20 Hz or 40 Hz). Resting state EEG (eyes open and closed) was recorded for 5 min before and after stimulation. At the end of each stimulation session, participants completed a questionnaire to evaluate the somatosensory effects induced by tACS. (B) Each trial of the backward masking task began with a set of four points moving to draw the attention of the participant to the fixation cross. Next, the target numeral briefly appeared in the display and was followed by a mask appearing at the past location of the target. The separations between target and mask (SOA) were uniformly spaced and randomly changed across trials.

A metacontrast backward-masking (Del Cul et al. 2007b) paradigm was implemented in PsychoPy v3.0 (Peirce et al. 2019). In each trial of this task, a target stimulus (consisting of the numerals 2, 3, 7, or 8) was followed by a mask with a variable temporal separation from the target, known as stimulus onset asynchrony (SOA; see Fig. 1a). Both mask and targets were randomly presented either to the left or to the right of a fixation cross on the 13-inch display of a MacBook Pro Retina (late 2013 model) with a refresh rate of 60 Hz. Following the presentation of the masked target, two forced-choice questions were presented in the screen. First, participants had to indicate whether the target was larger or smaller in magnitude than the numeral 5, thus providing an objective visibility measure. Second, participants had to report whether they saw the target numeral, which constitutes a subjective visibility measure. The complete task consisted of 400 trials with evenly distributed SOAs (16.7, 33.3, 50, 66.7, and 83.3 ms). Sixty randomly distributed trials lacked targets before the mask to control for subject engagement. The subjective contrast visibility threshold was determined in a prior separate session for each participant (see Supplementary Methods, *Determination of the subjective visibility threshold*) and implemented in the following three separate experimental sessions (sham, 20 Hz tACS, and 40 Hz tACS, see Supplementary Methods, *Experimental sessions*).

### Protocol for transcranial alternating current stimulation

Beta- (20 Hz), gamma- (40hz) and sham-tACS were applied using two hybrid tCS and EEG electrodes with a circular Ag/AgCl contact area (diameter: 12 mm; 8-channel Neuroelectrics STARSTIM dual EEG-tACS). The electrodes were placed at the Oz and Fz locations of the standard 10–10 EEG system. An intensity of 1 000 μA (2000 μA peak-to-peak) with a ramp-up of 20 s was applied. During sham-tACS, a frequency of 30 Hz was applied and discontinued after the ramp-up phase. Participants underwent tACS while they performed the metacontrast backward-masking task (duration ~20 min). The stimulation conditions were randomized and uniformly distributed throughout sessions and across participants. The randomization was performed by an individual not involved with the experiment, and the order of the stimulation conditions was stored in a spreadsheet that could be accessed by the researchers only at the data analysis step; thus, both participants and researchers were blind with respect to the applied stimulation.

### Questionnaires of somatosensory effects

After each session, participants completed a questionnaire assessing subjective sensations commonly associated with tACS stimulation. This questionnaire was included to assess the potential somatosensory effects induced by tACS, such as tingling or itching sensations, which could influence participants’ performance and interpretation of stimulation-related effects. This is particularly important given the ongoing debate about whether tACS effects are mediated by neural entrainment or peripheral sensations. The questionnaire ratings of somatosensory effects induced by tACS were normalized to scores ranging from zero to three for each sensation, with zero corresponding to feeling nothing, and three to feeling the sensation with strong intensity. These numerals were averaged for each subject to obtain one score for each condition.

### E‌EG recording

EEG was recorded in half the participants (Data collection had to be discontinued for the remaining participants due to an unexpected hardware failure that resulted in complete signal loss across all EEG channels.) (*n* = 17) before and after each tACS session using an 8-channel Neuroelectrics STARSTIM dual EEG-tACS. Electrodes were placed at stimulation sites (Fz, Oz) and additional locations (T3, T4, P3, P4, F7, and F8) following the 10–10 EEG system with Ag/AgCl electrodes. Ground and reference electrodes were positioned on the earlobe. Data were sampled at 500 Hz with 24-bit AD conversion and streamed *via* Bluetooth to a notebook. As the setup included only eight EEG channels, spatial resolution was limited. Therefore, topographical representations should be interpreted with caution.

### E‌EG processing and spectral analysis

EEG data were acquired and preprocessed as previously reported (Pallavicini et al. 2021; Tagliazucchi et al. 2021; Cavanna et al. 2022), using the EEGLAB Matlab toolbox (The MathWorks 2018). Signals were band-pass filtered (1–90 Hz) and notch filtered (47.5–52.5 Hz). Flatline channels (10x lower standard deviation) were removed, and artifacts were corrected using the “clean_rawdata” EEGLAB plugin (https://github.com/sccn/clean_rawdata). Rejected channels were interpolated, re-referenced to an average reference, and segmented into 2-s epochs. Major artifacts exceeding ±500 μV were removed, followed by rejection of noisy epochs using standard deviation thresholds (6 SD for single channels, 2 SD for all channels). Independent component analysis (ICA) was performed to identify and remove non-neural artifacts, including eyeblinks, muscle activity, cardiac artifacts and other non-neural activations. Components were visually inspected in EEGLAB based on their topographies, spectral profiles, and temporal dynamics. The number of ICs was limited to the retained channels using principal component analysis (PCA) to prevent rank deficiency.

Spectral power was computed using fast Fourier transform (FFT) on EEGLAB. For each epoch, the entire trial length (2 s) was used as the analysis window, and a Hanning taper was applied prior to transformation. No overlap between segments was used, and FFT points were within the 1–90 Hz range after applying a 1–90 Hz band-pass and a 47.5–52.5 Hz notch filter to suppress line noise. The DC component was removed before the computation, and power values were averaged linearly across trials before logarithmic conversion. The resulting log-transformed power spectra were then used for subsequent group-level analyses. We first examined power changes averaged across all electrodes to assess potential global effects of stimulation. This approach allowed us to detect broad modulations, which we then followed up with electrode-wise analyses to identify specific topographical effects. Differences were analyzed by subtracting baseline EEG (pre-tACS) from post-tACS recordings. Power changes at 20 Hz and 40 Hz were assessed separately for EO and EC conditions, yielding four comparisons (20 Hz-EO, 20 Hz-EC, 40 Hz-EO, 40 Hz-EC).

### Statistical analysis

Statistical analyses were conducted using the R (R Core Team 2014). Objective and subjective visibility were assessed as binary outcomes: objective visibility (“correct”/“incorrect”) was based on comparison with numeral 5, while subjective visibility (“seen”/“unseen”) depended on reported perception. Blindsight rate was the proportion of “correct” and “unseen” trials, while sightblind rate (the inverse) was the proportion of “incorrect” and “seen” trials.

Mixed logistic regressions tested differences in visibility rates (Bliksted et al. 2017; Chang et al. 2017; Łukowska et al. 2018)**,** modeling response measures as functions of tACS condition, session number, and SOA, with subject ID, target location, and numeral as random effects. Likelihood ratio tests compared full models to reduced models (without tACS condition) to assess tACS effects. Interaction models (tACS × SOA) were also evaluated.

Statistical significance of model coefficients was assessed *via* ANOVA (logistic models) or t-tests (linear models) using Satterthwaite’s method (α = 0.05). AUC (area under the curve) of visibility versus SOA was computed via the trapezoidal method:


$$ {\int}_a^bf(x) dx\approx \frac{1}{2}{\sum}_{n=1}^N\left({x}_{n+1}-{x}_n\right)\left[f\left({x}_n\right)+f\left({x}_{n+1}\right)\right] $$

where $a={x}_1<{x}_2<\dots <{x}_N<{x}_{N+1}=b$ and ${x}_{n+1}-{x}_n$is the spacing between each consecutive pair of points. Differences in AUC were tested using a linear model with tACS condition and session number as fixed effects, and subject ID as a random effect.

Questionnaire responses assessing somatosensory sensations during stimulation (e.g. itching, burning, and flickering) were analyzed using a linear model to describe the relationship between these scores and the tACS condition; specifically, tACS and session numbers were included as fixed effects and subject IDs as random effects. Correlations between questionnaire scores and behavioral performance were tested using Spearman’s rank correlation (ρ).

For the EEG data, spectral power was analyzed on artifact-free 2-s epochs. A 2×2 repeated-measures ANOVA was conducted with within-subject factors stimulation frequency (20 Hz, 40 Hz) and time (pre-, post-stimulation), separately for each condition (eyes open/closed). ANOVAs were run on both the channel-averaged spectral power and individual channels. To correct for multiple comparisons across electrodes, we applied the Benjamini-Hochberg False Discovery Rate (FDR) procedure with a significance threshold of α = 0.05. Post-hoc paired t-tests were used to assess differences between pre- and post-stimulation periods. Effect sizes were reported as partial eta squared (η^2^) or Cohen’s d where appropriate.

## Results

### Subjective and objective visibility as a function of stimulus onset asynchrony

**Figure 2:**
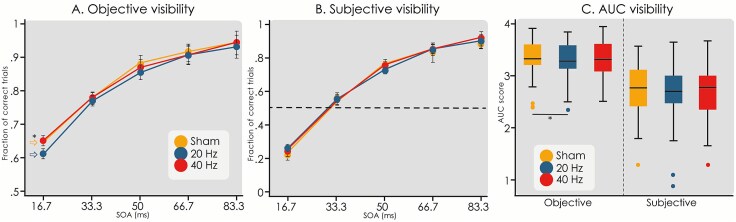
Objective and subjective visibility between tACS conditions. The target visibility rate as a function of SOA with error bars representing standard errors of the rates. (A) The objective visibility curve during 20 Hz-tACS was significantly lower compared to sham-tACS (Z = −4.16, *P =* 3.17e-05). No difference between 40 Hz-tACS and sham-tACS was found (Z = -1.61, *P* = .12). (B) Subjective visibility. The striped line shows that the subjective visibility threshold lies slightly below a SOA of 33.3 ms. No differences between tACS conditions were found for subjective visibility (χ2(2) = 0.7, *P* = .7). (C) The AUC scores for each tACS condition for the objective and subjective visibility curves. For the objective visibility, the AUC value for 20 Hz-tACS is significantly lower than the value obtained for sham-tACS (t = 2.42, *P* = .018).

Figure 2a and b show the objective and subjective visibility ratings as a function of SOA for all stimulation conditions. It is clear that both visibility measures increase with SOA. Indeed, SOA significantly affected the objective (*Z* = 44.18, *P* = 2e-16) and subjective (*Z* = 75.08, *P =* 2e-16) visibility. We observed significant session effects, particularly in sessions 3 (*Z* = 3.24, *P =* .001) and 4 (*Z* = 6.00, *P =* 2e-9) for objective visibility and session 4 (*Z* = 7.79, *P =* 7e-15) for subjective visibility, suggesting a learning-related increase in visibility measures. However, session was included as a fixed effect in the statistical models and session order was counterbalanced across participants, minimizing its influence on the interpretation of stimulation effects. Specifically, for each unitary increase in SOA the odds of a “correct” and a “seen” trial changed 1.78 (95% CI: 1.73, 1.82) and 2.69 (95% CI: 2.62, 2.76) for objective visibility and subjective visibility, respectively. Even though we performed a staircase session to determine an appropriate personalized contrast at which ~50% of the targets were visible for a SOA of 50 ms, Fig. 2b shows that the 50% subjective visibility threshold (averaged across participants) lies in the proximity of a SOA of 33.3 ms. Furthermore, the shape of the target visibility as a function of SOA did not exactly match previously reported findings (see Supplementary Fig. 1 of (Del Cul et al. 2007b)) since instead of a sudden increase in visibility, our data presented a more gradual increase (Fig. 2a).

### Effects of tACS stimulation

In order to fully characterize the response, we performed likelihood ratio tests to assess the variables included in the mixed logistic model (see methods). For the objective visibility rates, this test showed that the interaction between tACS condition and SOA was not significant (χ2(2) = 0.3, *P =* .86) and therefore was not included in the final model. An additional likelihood ratio test showed that tACS condition significantly affected the objective visibility rates (χ2(2) = 17.63, *P =* .00015) and the odds ratio of a “correct” trial for 20 Hz-tACS compared to sham-tACS was 0.84 (95% CI: 0.78, 0.91) (ANOVA with Satterthwaite’s method, *Z* = −4.16, *P =* 3.17e-05). In other words, the probability of a “correct” trial decreased during 20 Hz-tACS compared to sham-tACS. Furthermore, the odds ratio of a “correct” trial for 40 Hz-tACS compared to sham-tACS was not significant (odds ratio = 0.94, 95% CI: 0.86, 1.01;*Z* = -1.61, *P =* .12). The AUC of the objective visibility as response to SOA was computed for each tACS condition (Fig. 2c). A one-sided likelihood ratio test showed that tACS condition significantly affected the AUC (χ2(2) = 5.67, *P =* .029). In line with the previous results, AUC decreased during 20 Hz-tACS compared to sham-tACS (decreased by −0.073, 95% CI: −0.13, −0.013, *t* = −2.42, *P =* .018).

For the subjective visibility rates (Fig. 2b), the data showed no significant interaction between tACS condition and SOA (χ2(2) = 5.57, *P =* .062) and therefore this interaction was not included in the final model. Moreover, an additional likelihood ratio test showed that tACS condition did not significantly affect the subjective visibility rates (χ2(2) = 0.7, *P =* .7). Consistently, tACS condition did not affect the computed AUC for the subjective visibility as response to SOA (χ2(2) = 0.23, *P =* .89), as shown in Fig. 2c.

**Figure 3:**
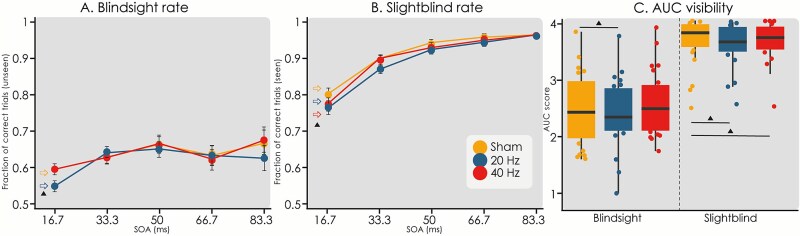
Effect of 20 Hz and 40 Hz tACS on blindsight and Sightblind rates compared to sham. (A) The blindsight rate (correct but unseen trials) for 20 Hz-tACS was lower compared to sham-tACS (Z = 2.57, *P* = .01). (B) The sightblind rates (seen but incorrect trials) of both 20 Hz-tACS (Z = −3.6, *P* < 0.001) and 40 Hz-tACS (*Z =* −2.22, *P* < .05) were lower compared to sham-tACS. (C) Area Under the Curve (AUC) for the confidence ratings displayed in panels A and B, summarizing changes across SOAs for each tACS condition. A triangle (▲) indicates a significant main effect of condition as revealed by the full statistical model, although *post hoc* pairwise comparisons of AUCs were not significant. This plot is provided for illustrative purposes, to visualize the direction of the modeled effect.

To determine whether the decrease in objective visibility reflected a decrease in subliminal processing, we additionally analyzed blindsight rates (Fig. 3a), this rate refers to the proportion of trials in which participants correctly categorized the stimulus but reported not having seen it (typically interpreted as subliminal processing). In this case, the interaction between tACS condition and SOA was not significant (χ2(2) = 0.91, *P =* .63) and therefore not included in the final model. The final model showed that the odds of a blindsight trial significantly increased with 1.14 (95% CI: 1.1, 1.18) for each unitary increase in SOA (*Z* = 7.211, *P =* 5.53e-13; see Fig. 3A). Moreover, the data showed that tACS condition significantly affected the blindsight rate (χ2(2) = 6.79, *P =* .034). Specifically, the odds of a blindsight trial for 20 Hz-tACS compared to sham-tACS was 0.88 (95% CI: 0.79, 0.97), or in other words, the probability of a blindsight trial decreased during 20 Hz-tACS (*Z* = 2.57, *P =* .01). The 40 Hz-tACS coefficient was not significant, meaning that there was no difference in blindsight rates between 40 Hz-tACS and sham-tACS (odd ratio: 0.96, 95% CI: 0.86, 1.06, *Z* = −0.89, *P =* .38).

The sightblind rate was analyzed to verify whether the decrease in blindsight rate actually represented a decrease in subliminal processing or was a result of a general decrease in objective visibility (Fig. 3b). The sightblind rate reflects trials in which participants reported seeing the stimulus but categorized it incorrectly, often interpreted as reflecting low-confidence or overconfident errors. The data showed that tACS condition also affected sightblind (χ2(2) = 13.11, *P =* .0014), with an odds ratio of 0.75 (95% CI: 0.64, 0.86) for “correct”; and “seen” trials during 20 Hz-tACS compared to sham-tACS (*Z =* −3.6, *P =* .00032). This result suggests that the decrease in blindsight is not specific to subliminal processing but an effect of a general decrease in objective visibility. Interestingly, the odds ratio of a sightblind trial during 40 Hz-tACS was 0.83 (95% CI: 0.71, 0.98) and significant as well (*Z =* −2.22, *P =* .026). Moreover, similarly to the previous results, SOA significantly affected sightblind (odd ratio: 0.78, 95% CI: 1.69, 1.87, *Z =* 22.97, *P =* 2e16). The reaction times (RTs) for the objective response were analyzed to determine whether they were related to the decrease in objective visibility. Pearson’s correlations between the proportion of correct trials (objective visibility) and RTs were performed; however, no significant correlations were found (*r* = 0.11, *P =* .28).

**Figure 4:**
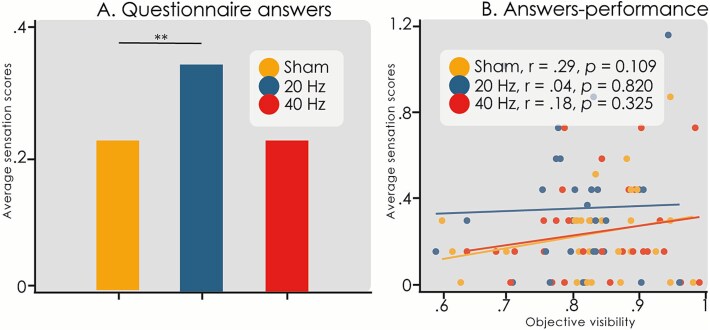
Questionnaire scores. (A) Average somatosensory sensation scores per stimulation condition (^*^^*^P < .01). (B) Scatter plot of average somatosensory sensation scores versus objective visibility across conditions. No significant correlation was found between these variables.

Next, we analyzed the questionnaire ratings to determine whether subjects were able to distinguish between tACS conditions based on the somatosensory perceptions they experienced. Interestingly, tACS condition significantly affected the somatosensory scores (χ2(2) = 9.22, *P =* .01), increasing the total score by 0.11 (95% CI: 0.03, 0.33) during 20 Hz-tACS compared to sham-tACS (*t* = 2.721, *P =* .0085, Fig. 4a). In contrast, no difference in scores between sham-tACS and 40 Hz-tACS was found (*t* = 0.009, *P =* .99). Since 20 Hz-tACS decreased the objective visibility as well as increased the questionnaire scores, a Pearson’s correlation test between these response measures was performed to investigate a possible relationship between both effects; however, no correlation was found (r = .04, *P =* .820) (Fig. 4b). Additionally, no significant correlations were found between Sensation scores and objective visibility in the Sham condition (*r* = .29, *P =* .109) or the 40 Hz tACS condition (*r* = .18, *P =* .325) (Fig. 4b).

### E‌EG spectral power

**Figure 5:**
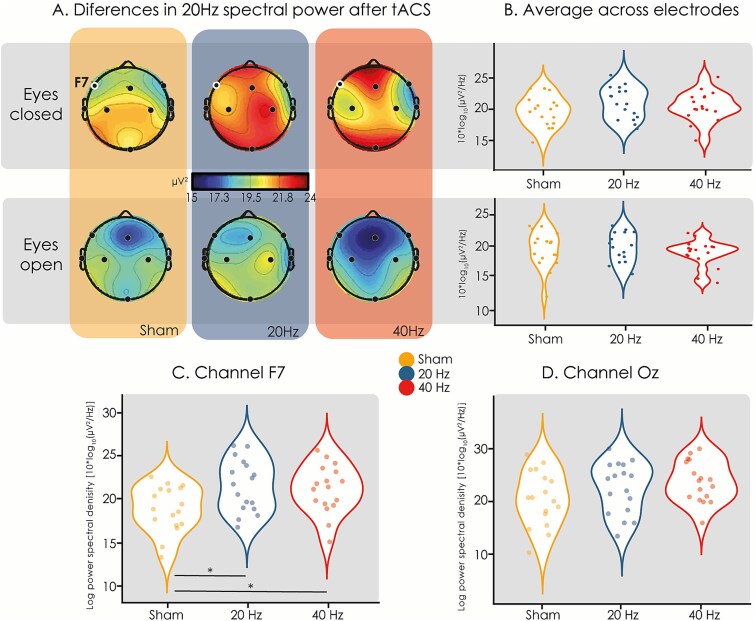
Differences in 20 Hz spectral power between tACS conditions. (A) Baseline EEG spectral power was subtracted from post tACS EEG. Both 20 Hz-tACS and 40 Hz-tACS increased 20 Hz power compared to sham-tACS during the EC condition (top row panels). This increase was only significant for channel F7 (highlighted dot). In contrast with the EC (top row panels) condition, no significant spectral power differences were found for the EO (bottom row panels) condition. Due to the limited number of EEG channels, scalp maps should be interpreted with caution and do not allow precise localization of neural activity. (B) Differences in log power spectral density between tACS conditions for all averaged electrodes. No significant differences were found. (C) Results for frontal channel (F7). Notice that both 20 Hz-tACS (t(16) = −3.17, *P* = .048) and 40 Hz-tACS (t(16) = −3.17, *P* = .029) show higher 20 Hz power compared to sham-tACS. (D) Results for occipital central channel (Oz). Increased 20 Hz power during the EC condition showed a tendency towards significance (*F*(2,15) *=*4.99, *P* = .0524).

**Figure 6:**
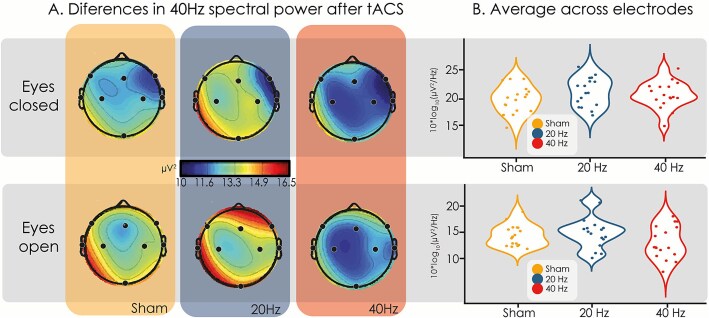
Differences in 40 Hz spectral power between tACS conditions. (A) Scalp distribution of post-stimulation EEG spectral power minus baseline (pre-stimulation) for each condition. Although visual inspection of the topographies suggests a trend toward reduced 40 Hz power in the 40 Hz-tACS condition (more blue) compared to sham, no statistically significant differences were found. Due to the limited number of EEG channels, scalp maps should be interpreted with caution and do not allow precise localization of neural activity. (B) Averaged spectral power across all electrodes grouped by tACS stimulation condition.

Figures 5 and 6 present the differences in 20 Hz and 40 Hz power for each tACS condition, respectively, before and after the metacontrast backward-masking task. The topographic plots suggest that 20 Hz power increased during the EC condition after 20 Hz-tACS (see Fig. 5a). Although this was not the case for the averaged power across all electrodes (Fig. 5b), a significant power difference at 20 Hz was observed in channel F7 (F(2,15) = 6.43, *P =* .0029, FDR corrected; Fig. 5c). Both the 20 Hz-tACS (*t*(16) = −3.17, *P* = .048) and 40 Hz-tACS (*t*(16) = −3.17, *P =* .029) conditions increased 20 Hz power compared to sham-tACS (see Fig. 5c). No other channels increased 20 Hz power during the EC condition, although Oz showed a tendency towards significance (*F*(2,15) *=*4.99, *P =* .0524) (Fig. 5d). The 20 Hz power differences during the EO condition were less pronounced (Fig. 5a). Only after the 40 Hz-tACS condition a decrease in 20 Hz spectral power was apparent; however, no statistically significant differences were found for 20 Hz power during EO. Figure 6 presents the differences in 40 Hz power between tACS conditions. No significant differences were found in 40 Hz power after 40 Hz-tACS with both the EO and EC.

## Discussion

We implemented a tACS protocol to investigate the causal relationship between beta and gamma oscillations and conscious information access. Based on prior studies and theoretical considerations, we hypothesized that beta-tACS would affect subjective visibility of visual targets due to its link to conscious processes (Lamme and Roelfsema 2000) (Engel et al. 2001; Sergent and Dehaene 2004b; Melloni et al. 2007b; Gaillard et al. 2009b; Steinmann et al. 2014; Cavinato et al. 2015; Bastos et al. 2015b). Our results offer partial support for our original hypotheses. Contrary to our expectations, beta tACS reduced objective visibility without affecting subjective visibility. However, we also observed decreased both blindsight (correct but unseen trials) and sightblind rates (seen but incorrect trials). We did not find significant effects for 40 Hz tACS. Blindsight relates to subliminal processing, while sightblindness is linked to confidence (Sergent and Dehaene 2004c). Since both measures were affected, identifying the specific neural mechanism remains unclear. To explore potential confounds, we also analyzed post-session questionnaires to assess somatosensory sensations during stimulation. Participants reported increased discomfort during beta-tACS, which may have influenced their perceptual and cognitive performance more generally. We believe that collecting participants’ perceptual experiences during stimulation is essential when investigating subjective awareness and should become standard practice in similar experimental designs.

The primary result of our study is the decrease in objective visibility under beta-tACS. However, the mechanisms by which oscillatory electrical fields interact with neural activity remain poorly understood. While our experiment does not involve learning or memory consolidation, we briefly reference tACS studies during NREM sleep to illustrate the broader context of how rhythmic stimulation can modulate neural processes. For instance, low-frequency tACS during NREM sleep enhances memory presumably by entraining these nested rhythms (Marshall et al. 2006; Antonenko et al. 2013; Binder et al. 2014; Prehn-Kristensen et al. 2014; Westerberg et al. 2015; Ladenbauer et al. 2017). More generally, there is evidence that tACS can entrain endogenous rhythms in gamma (Reato et al. 2010; Strüber et al. 2014; Voss et al. 2014), beta (Pogosyan et al. 2009), alpha (Zaehle et al. 2010; Merlet et al. 2013), theta (Marshall et al. 2011), and delta bands (Marshall et al. 2006; Fröhlich and McCormick 2010; Ozen et al. 2010). However, replication has been inconsistent, with studies reporting no significant behavioral or physiological effects (Brignani et al. 2013; Eggert et al. 2013; Sahlem et al. 2015; Horvath et al. 2015a; Horvath et al. 2015b). In a recent study, Lafon et al. showed that low-frequency tACS (of 2.5 mA intensity) was unable to modulate spindle and gamma dynamics during NREM sleep (Lafon et al. 2017; Lafon et al. 2018). Following a different approach, a recent review claims that stimulation with ~1 mA peak intensity is able to induce only 0.1–0.2 mV changes in membrane potential (Liu et al. 2018a). This raises the possibility that some behavioral effects observed in tACS studies may not arise from direct cortical modulation, but rather from peripheral nerve stimulation.

To assess whether beta-tACS effects were related to changes in beta power, we analyzed EEG spectral power differences pre- and post-tACS. Our results showed increased beta power after both beta- and gamma-tACS, suggesting a nonspecific effect rather than targeted beta entrainment. A plausible explanation is that tACS could have stimulated subcutaneous nerves, which in turn signaled to the brain and changed EEG power (Liu et al. 2018b). Significant beta power differences appeared only in one frontal channel during the eyes-closed condition, suggesting that if beta oscillations were induced, the effect was localized. While our topographical findings suggest differential spectral power patterns following stimulation, these results are based on a low-density montage and should therefore be interpreted with caution. Higher-density EEG would be required to confirm and refine these spatial patterns.

Research suggests that long-range synchrony for conscious access is not easily achieved at high frequencies (Fries 2005; Lachaux et al. 2005; Buschman and Miller 2007; Buzsáki 2009), proposing synchrony in the beta range as the main contributor to this phenomenon. Thus, reduced target visibility under 20 Hz tACS may be due to interference with synchronizing activity rather than increased beta power. In line with this, Helfrich et al (Helfrich et al. 2014) showed that while in-phase interhemispheric 40 Hz-tACS enhanced parieto-occipital synchronization and the associated perceptual correlates, anti-phase stimulation reduced it. Also, we cannot rule out the possibility that our tACS protocol only induced small local increases in beta power but was not capable of establishing inter-areal beta synchrony, a possibility that could be investigated using high density EEG combined with source modeling and measures such as phase transfer entropy or granger causality. In future experiments, it might also be interesting to apply closed-loop tACS to modulate brain activity in a phase-dependent manner.

During beta-tACS, subjects reported increased somatosensory sensations, including tickling, burning, and visual flickering, compared to sham-tACS. This supports the possibility that behavioral differences stemmed from non-neural effects of tACS, potentially distracting subjects and impairing task performance. Previous studies using similar questionnaires established that tACS-induced sensations are frequency-dependent, with 20 Hz being the most perceptible (Turi et al. 2013). Since studies suggest subjects cannot distinguish active versus sham tACS (Wach et al. 2013a; 2013b), unblinding is unlikely, but sensory intrusion remains a concern. However, no correlation was found between questionnaire scores and performance. Importantly, while this kind of questionnaire is seldomly used in studies using tACS or other forms of stimulation, we have shown that it can provide useful information for the interpretation of the experimental results. Given the variability in individual responses to stimulation and the potential influence of somatosensory sensations, our results should be interpreted with caution, and future studies should systematically control for these factors.

Our study showed that beta-tACS, but not gamma-tACS, influenced visual perception, indicating frequency-specific effects. Most EEG studies focus on gamma oscillations in conscious perception, but our findings suggest that beta oscillations may play a more central role. While many studies link gamma power and synchrony to resolving ambiguous stimuli (Laczó et al. 2012; Helfrich et al. 2014; Cabral-Calderin et al. 2015) gamma may be more involved in feature binding than conscious access. Nunez & Srinivasan (Nunez and Srinivasan 2010) argued that gamma oscillations are overemphasized in studies of consciousness, while lower frequencies like beta are better suited for large-scale synchrony (Kopell et al. 2000; von Stein and Sarnthein 2000; Buzsáki and Draguhn 2004). Importantly, studies of the visual cortex showed that gamma oscillations travel in a feedforward direction, while lower frequencies, such as beta and alpha, travel in a feedback direction (van Kerkoerle et al. 2014b; Bastos et al. 2015b), and thus they might play a fundamental role in conscious perception, insofar it necessitates top-bottom feedback for the amplification of the incoming sensory information.

Beyond the beta-gamma dichotomy, neural oscillations likely operate through complex interdependencies rather than isolated frequency-specific functions. There is a marked tendency to associate well-defined cognitive functions to specific oscillatory frequencies; however, it is unlikely that oscillations can be mapped into completely separate functions. Moreover, it has been shown that different frequencies present mutual interactions, such as when fast frequencies are nested on slower ones (Roopun et al. 2008; Belluscio et al. 2012). Thus, identifying feedforward and feedback activity solely with gamma and beta bands may be oversimplified. Even if this is not the case, strong mutual interdependences between cortical oscillations could impede the selective empirical manipulation of narrow frequency bands by external means, as we attempted in the present study.

Interestingly, our results did not show the nonlinear increase in visibility as a function of SOA, as shown by Del Cul and colleagues (Del Cul et al. 2007b). This may be due to our use of a personalized target contrast during staircase calibration, which likely resulted in an overall easier task for participants. The objective of including a personalized contrast was to control for inter-subject variability, setting a common visibility threshold at a SOA of 50 ms. However, the resulting visibility threshold was considerably lower, therefore, it might be possible that on average the contrast was too high and consequently that the task was too easy for the participants. In this case, the sudden increase in visibility would not be found because the target numerals were already visible at the lower SOAs. Alternatively, conscious perception may occur more gradually than predicted by the GW model (Sergent and Dehaene 2004b), aligning with theories proposing a continuum of activation strength (Nieuwenhuis and de Kleijn 2011). Theoretical considerations aside, the way the subjects understand the instructions to report and judge what constitutes conscious perception for them is likely to influence the nonlinearity of the subjective visibility curve. As with somatosensory questionnaires, debriefing participants on their subjective experience could clarify discrepancies.

## Summary and conclusions

In summary, this study showed that beta-tACS is capable of modulating target visibility in a metacontrast backward-masking task. While interesting and novel, it is not yet certain that this modulation is mediated by an effect of tACS on neural activity. Future studies should examine inter-areal connectivity changes following beta-tACS to better understand its effects. The tACS literature contains contradictions, with some studies suggesting higher intensities (>2.5 mA) may be necessary for neural entrainment, yet entrainment is still cited as its primary mechanism. Alternative approaches, such as in-phase stimulation in closed-loop setups, could enhance resonant effects (Ketz et al. 2018), a possibility to be explored in future experiments. Finally, even under optimal conditions, the selective manipulation of a frequency band by noninvasive electrical stimulation could be difficult or even impossible, highlighting the need to back up our conclusions with further work in animal models, in combination with computational studies capable of exploring the mechanistic causes and implications of our findings.

Importantly, our results also underscore the relevance of somatosensory effects during stimulation, a factor rarely addressed in tACS studies. These sensations may meaningfully impact perceptual and cognitive outcomes, and their systematic consideration could improve the interpretability and methodological rigor of future noninvasive brain stimulation research.

## Supplementary Material

Supp_tACS-visibility_niaf056

## Data Availability

All data and codes are available upon request to authors.
