# Supplementary material for: Investigating the role of beta and gamma tACS in visual processing and conscious perception
Source: Neurosci Conscious. 2026 Jan 19;2026(1):niaf056. doi: 10.1093/nc/niaf056 (PMC12814965; doi:10.1093/nc/niaf056)
Supplement: Supp_tACS-visibility_niaf056 [file supp_tacs-visibility_niaf056.docx]

**Investigating the Role of Beta and Gamma tACS in Visual Processing and Conscious Perception**

Yayla A. Ilksoy^1,*^, Alethia de la Fuente^1,2,3,*^, Jacobo Diego Sitt^4^, Enzo Tagliazucchi^1,2,5^, Carla Pallavicini^1,2,6^

1. Department of Physics, University of Buenos Aires, Argentina.

2. National Scientific and Technical Research Council, Buenos Aires, Argentina

3. Institute of Cognitive and Translational Neuroscience, INECO Foundation, Favaloro University

4. Sorbonne Université, Institut du Cerveau - Paris Brain Institute - ICM, INSERM, CNRS, Paris, France

5. Latin American Brain Health Institute (BrainLat), Universidad Adolfo Ibanez, Santiago, Chile

6. Cognitive Neuroanatomy Lab, INCC UMR 8002, CNRS, Université Paris Cité, Paris, France

* Both authors contributed equally to this work

**Supplementary Methods**

*Determination of the subjective visibility threshold*. A staircase procedure was implemented to determine a stimulus-background contrast for which each participant achieved subjective visibility in ~50% of the trials at the SOA of 50 ms. The contrast of the numeral presented as the masked stimulus could linearly range from -1 (gray, background color) to 1 (black). At the beginning of the staircase session, the contrast was set to 0.7 and could increase or decrease in steps of 0.2, depending of the response given by the participant at each trial. The contrast decreased when the objective response was correct and the subjective visibility rating was affirmative, and increased when the subject either incorrectly compared the stimulus to 5, or declared absence of subjective visibility. The final contrast used for the experimental task was computed as the mean of the last eight reversals of the staircase session.

*Experimental sessions.* Participants performed a total of four sessions on four non-consecutive days (average days between sessions: 6.14 $\pm$ 3.15, between 1 and 15 days). On the first session, participants performed a short training followed by the staircase. On the second, third and fourth sessions participants performed the metacontrast backward-masking task, while being stimulated with either 20Hz-, 40Hz or sham-tACS (counterbalanced and double-blind). Furthermore, preceding and following each tACS session, resting state EEG was recorded for 5 min with eyes closed (EC-EEG) and with eyes open (EO-EEG; see Figure 1B). Participants performed the sessions approximately at the same time of the day as in their previous sessions (average hour difference between sessions: 2:03). All experimental sessions were performed in a darkened and acoustically isolated room. At the end of each session with tACS stimulation, subjects completed a standardized questionnaire [30] to assess the somatosensory effects elicited by the stimulation (e.g. itching, burning).

**Supplementary results**

**
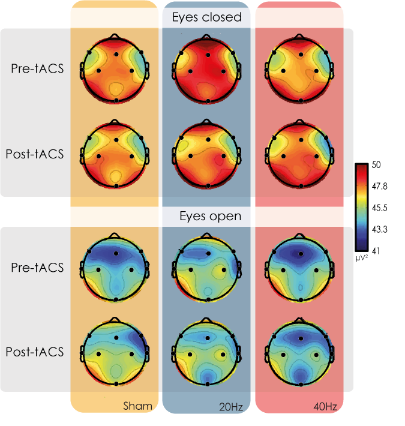
**

**Supplementary figure 1:** Same EEG topographies as in figure 5 (20Hz power differences) but without subtracting baseline EEG (pre-tACS) from post-tACS EEG.

**
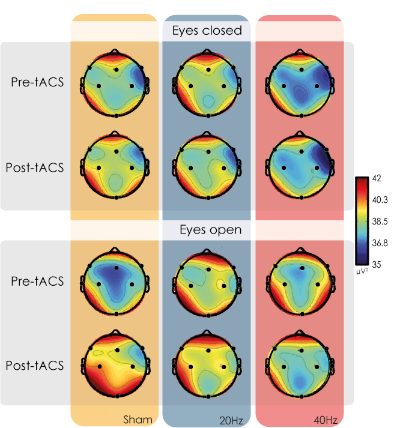
**

**Supplementary figure 2:** Same EEG topographies as in figure 6 (40Hz power differences) but without subtracting baseline EEG (pre-tACS) from post-tACS EEG.
